# Blood loss anemia due to adenocarcinoma of the jejunum: case report and review of the literature

**DOI:** 10.4076/1757-1626-2-6237

**Published:** 2009-02-18

**Authors:** João Figueira-Coelho, Sofia Lourenço, Michele Costa, Paula Mendonça, António Murinello, Jorge Neta

**Affiliations:** 1 Hospital de Curry Cabral - Department of Internal Medicine I - Rua da Beneficência nº8 1069-166 LisboaPortugal; 2 Hospital de Curry Cabral - Department of Pathology - Rua da Beneficência nº8 1069-166 LisboaPortugal

## Abstract

**Background:**

Small bowel tumors are rare, accounting for only 3-6% of gastrointestinal neoplasms, 1-2% of these being malignant. They must be considered whenever a patient presents with gastrointestinal bleeding, with normal upper gastrointestinal endoscopy and colonoscopy.

**Case presentation:**

We report a case of jejunal adenocarcinoma presenting as a blood loss anemia in a 65 year-old male, doing a brief review on the subject.

**Conclusion:**

Our case intends to highlight the fact that small bowel tumours are rare and frequently present to the Internist as non-specific clinical symptoms.

## Background

Small bowel tumors are rare, accounting for only 3-6% of gastrointestinal neoplasms, 1-2% of these being malignant [1,2]. The diagnosis is usually made in an advanced stage due to non-specific clinical symptoms [3]. In up to 5% of gastrointestinal bleedings the source remains unidentified after upper and lower endoscopic examination. In these cases, one must consider lesions in the small bowel, being vascular ectasias and tumors the most common causes.

## Case presentation

A 65 year-old white male began symptoms of weakness for medium efforts in March 2007. Two months later, during one week, he had 2 episodes of dark feces, associated with periumbilical pain, irradiating to the flancs and epigastric area. He denied heart burn, pyrosis, regurgitation, post-prandial fullness, nausea, vomiting, diarrhea and loss of apetite. Blood tests revealed normocytic normochromic anemia (hemoglobin = 11.1 g/dL), and upper gastrointestinal endoscopy was normal. During the next two months there was aggravation of anemia (hemoglobin = 7.2 g/dL, mean globular volume = 67.6 fL) and progressive asthenia, without evident blood loss or gastrointestinal symptoms, coming then to the Emergency Room. The patient had a past history of chronic hepatitis B, arterial hypertension, gout, dislipidemia and had removed colo-rectal polyps, with an unknown histologic diagnosis, 3 years before. He presented an alcohol consumption of 75 g of ethanol / day, was a former smoker (10 pack years), and was medicated with losartan+hydrochlorothiazide, allopurinol and simvastatin+ezetimibe. In the past 18 months the patient referred a weight loss of 15 kg.

**Figure 1.**
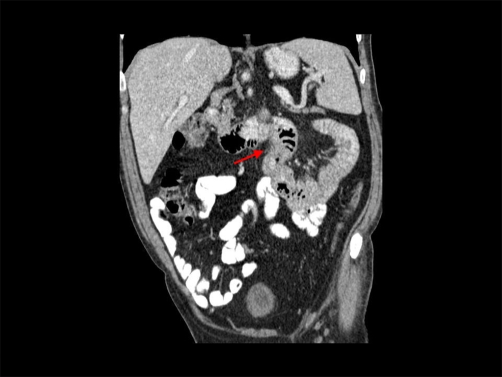
Diagnostic CT scan view showing diffuse upper jejunal thickening.

**Figure 2.**
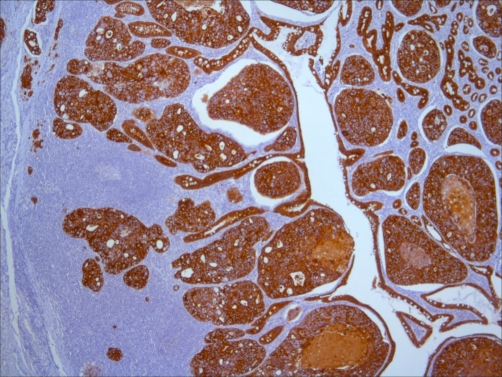
Neoplasic cells stained with AE1/AE3, a pan-keratin marker (40x), showing its epithelial origin.

**Figure 3.**
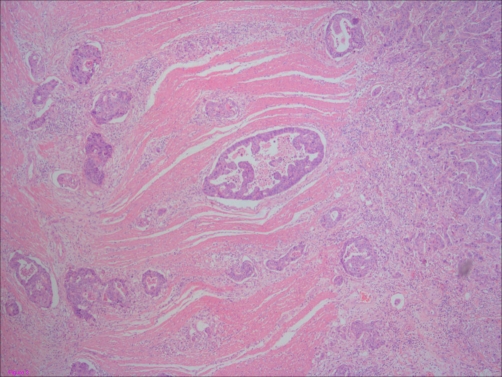
The infiltrative character of this tumour presented here with invasionof the muscularis propria (Hematoxilin & Eosin, 100X).

Physical observation, apart from descoloration of skin and mucous membranes, was unremarkable, revealing no palpable adenomegaly (including absence of Virchow node), no abdominal masses, and a normal digital rectal examination. Blood tests confirmed a blood loss / iron-deficiency anemia (serum iron = 4μg/dL, serum ferritin = 8.1 ng/dL, percent transferrin saturation = 1.06%, total iron-binding capacity = 313 mg/dL), but were otherwise normal. Chest x-Ray showed cardiomegaly and clear lungs. The upper gastroduodenal endoscopy revealed no lesions. Colonoscopy identified 3 polyps, with no signs of bleeding, in the sigmoid, descendent colon and cecum, corresponding to tubular adenomas with low-grade dysplasia which did not seem to be responsible for the clinical presentation. Since we were unable to proceed with capsule endoscopy due to difficult availability in our hospital, we decided to do an abdominal CT-scan, which showed a diffuse upper jejunal thickening with local, retrocural and peri-aortic adenomegaly, along with distension of D3 suggesting a suboclusive pattern (Fig. 1). The day after the CT scan, the patient began with retching, so programmed surgery was antecipated. Resection of upper jejunum and regional lymph nodes, with jejuno-duodenal anastomosis, was performed. The histologic examination revealed an ulcerated, poorly diferentiated adenocarcinoma - T4N2M0 (stage III) (Figs. 2 and 3).

Six months after surgery, follow-up CT scan revealed evidence of local recurrence of the tumour, again with lymph node involvement. The patient was proposed for chemotherapy with six cycles of capecitabine. By the end of the second cycle the patient started vomiting and abdominal pain relieved by metochlopramide and diazepam, allowing the third cycle. Due to recurrence of symptoms and marked weight loss he then repeated the CT scan, once more revealing diffuse upper jejunal thickening with distension of D3, extensive lymph node dissemination (abdomen and thorax), and a single hepatic metastasis at segment IV. A paliative bypass was performed with successful symptomatic relieve and the patient is still alive 14 months after diagnosis.

## Discussion

The small bowel corresponds to 75% of the digestive tract lenght and 90% of its mucosal surface area [4], but surprisingly the frequency of neoplasms is lower than in the stomach or the large bowel. This apparent “neoplastic resistance” finds its eventual explanation in the low bacterial content capable of transforming food components into carcinogens, along with the large amount of alcaline fluid secretion, the rapid transit time, and the high concentration of the enzyme benzopyrene hydroxylase, which reduce the mucosal contact with potencial carcinogens [5].

In 1746, Hamburger made the first case report of duodenal carcinoma, and the first review of malignant small bowel neoplasms was published, in 1876, by Leichtenstein.

In 2007, the American Cancer Society reported 5640 new cases of small bowel tumors, with 1090 estimated deaths [6]. A population-based study of the incidence of malignant small bowel tumors made in the United States, between 1973 and 1990, revealed an incidence rate per million people of 3.7 for adenocarcinoma and 3.8 for carcinoid, the 2 most common histologic types [5].

Data from Germany suggests an annual incidence of small bowel adenocarcinomas of 2.2-5.7 per million people each year [7].

The mean age of diagnosis of small bowel tumors is 60 years of age, with a slight male preponderance [1,2]. Adenocarcinoma is the most common histologic type (30-50%). The other epithelial origin variants, like mucinous adenocarcinoma or the small cell, signet-ring cell, squamous and adenosquamous, medullary or even undifferenciated are much rarer. The carcinoids (25-30%) follow the lead, and in a lesser extent are the lymphomas and sarcomas (which include gastrointestinal stromal tumors). The frequency of small bowel tumours is higher in the duodenum (38-55.2%), and decreases distally, with 17.6-33.3% cases localized in jejunum and 13-23.8% in the ileum [2,4,8]

Small and large bowel adenocarcinomas share similar pathologic (development from adenomatous polyps), genetic (mutations of oncogene *ras* and elevated levels of *p53*), and epidemiologic features [9]. Familial adenomatous polyposis, hereditary non-polyposis colorectal cancer, Crohn´s disease, celiac disease and diet (animal fat, read-meat and smoked/salted food) are the strongest risk factors [4].

The diagnosis is usually delayed in about 6 to 8 months due to nonspecific clinical symptoms. Abdominal pain (50-75%), nausea / vomiting (3372.5%), weight loss (38-52.5%), intestinal obstruction (31.3-44%), and gastrointestinal bleeding (23-33%) are the most common symptoms [1,2,10]. At the time of the diagnosis tumors are already in an advance stage [1,2]. In a review of 217 adenocarcinomas, made by Dabala *et al* [3], 39% of the patients were in TNM stage III and 35% in stage IV by the time of the diagnosis.

Capsule endoscopy has become the most sensible and specific diagnostic method for small bowel disease [11]. It has increased the diagnostic rate of small bowel tumors, most of them detected in cases of obscure gastrointestinal bleeding, with 50-60% of these being malignant. The capsule has overcome the low sensitivity of barium studies (30-50%), and the discomfort and complexity of push and sonde enteroscopy. The upper gastrointestinal endoscopy cannot pass beyond the Treitz ligament so, although it identifies around 93% of duodenal tumors, its overall sensitivity is only 31%. CT scan misses 43% of small bowel tumors [1].

Surgical resection is the only hope of cure for patients with small bowel adenocarcinoma. Pancreatoduodenectomy is performed in tumors of the first and second portion of the duodenum, while resection followed by anastomosis is the option for tumors located elsewhere. In each case, resection must include regional lymphadenectomy due to the high prevalence of lymph node metastasis at the time of presentation. Curative resection of small bowel tumors is obtained in about 50% [1]. In a recent review, made by Agrawal *et al* [10], curative resection for adenocarcinoma was performed in 54.5% of cases, with a 3.6% postoperative mortality rate. Despite curative surgery, the disease will eventually recur in half of the patients with lymph node, hepatic or lung metastasis.

Chemotherapy for small bowel tumors is still a matter of debate. There are no randomised controlled trials to evaluate the effectiveness of adjuvant chemotherapy for adenocarcinoma of the small intestine, and so no optimal chemotherapy regimen has yet been established. In 1984, Jigyasu *et al* [12] reported a small benefit in 3 of 21 patients with metastatic disease, and Ouriel and Adams [13] observed a small increment of survival in 6 patients with metastatic and 6 with recurrent disease, both studies based on the use of chemotherapy regimens containing 5-fluorouracil. In a study, by Crawley *et al* [14] in 1998, 3 of 8 patients with advanced diseased showed good response to the same chemotherapy agent. Agents used in colorectal carcinoma have also been used in small bowel adenocarcinoma. Polyzos *et al* [15] used irinotecam in 3 patients with disease refractory to 5-fluorouracil, obtaining minor responses and some improvement in symptoms. Some recent studies have failed to prove a clear benefit of adjuvant chemotherapy [3].

Radiotherapy does not seem to be effective or benefit survival. It is technically difficult to localize the target field due to the mobile nature of the small intestine mesentery.

The prognosis of small bowel tumors is poor, with most series reporting a 5-year-survival of 15-35%. After curative surgical resection this rate increases to 40-65% in most studies. In 1999, Howe *et al* [8] presented a review of 4995 cases of adenocarcinoma, and defined patient age over 75, duodenal localization, advanced stage and absence of surgery treatment as negative prognosis factors. Adenocarcinoma seems to be the second histologic type with the best suvival rate, as reported by Talamonti *et al* [2]. In this review 37% of the patients with adenocarcinomas were alive 5 years after the diagnosis with a median survival of 30 months, surpassed only by carcinoids (64%), and in contrast to sarcomas (22%) and lymphomas (29%). Median survival for incomplete resected adenocarcinomas was 9 months, increasing to 40 months, with a 5-year survival rate of 42%, for completely resected adenocarcinomas, which supports the positive prognostic value of surgery.

## Conclusion

Our case intends to highlight the fact that small bowel tumours are rare and frequently present to the Internist as non-specific clinical symptoms. Although capsule endoscopy has become the most sensible and specific diagnostic method for small bowel disease, the use of other diagnostic tools, like CT scan, are still a valid option where capsule is not easily available. Despite surgery and trial of chemotherapy the disease tends to recur conferring a poor prognosis to this rare gastrointestinal tumor.
